# Sarcopenia and Diabetes: A Detrimental Liaison of Advancing Age

**DOI:** 10.3390/nu16010063

**Published:** 2023-12-25

**Authors:** Giuseppe Lisco, Olga Eugenia Disoteo, Anna De Tullio, Vincenzo De Geronimo, Vito Angelo Giagulli, Fabio Monzani, Emilio Jirillo, Renato Cozzi, Edoardo Guastamacchia, Giovanni De Pergola, Vincenzo Triggiani

**Affiliations:** 1 Section of Internal Medicine, Geriatrics, Endocrinology and Rare Diseases, Interdisciplinary Department of Medicine, University of Bari “Aldo Moro”, 70124 Bari, Italy; annadetullio16@gmail.com (A.D.T.); vitogiagulli58@gmail.com (V.A.G.); emilio.jirillo@uniba.it (E.J.); edoardo.guastamacchia@uniba.it (E.G.); 2 Unit of Endocrinology, Diabetology, Dietetics and Clinical Nutrition, Sant Anna Hospital, 22020 San Fermo della Battaglia, Italy; olgaeugenia.disoteo.amediabete@gmail.com; 3 Unit of Endocrinology, Clinical Diagnostic Center Morgagni, 95100 Catania, Italy; vdg@iol.it; 4 Geriatrics Unit, Department of Clinical & Experimental Medicine, University of Pisa, 56126 Pisa, Italy; fabio.monzani@med.unipi.it; 5 Division of Endocrinology, Niguarda Hospital, 20162 Milan, Italy; dr.renatocozzi@gmail.com; 6 Center of Nutrition for the Research and the Care of Obesity and Metabolic Diseases, National Institute of Gastroenterology IRCCS “Saverio de Bellis”, 70013 Castellana Grotte, Italy; giovanni.depergola@irccsdebellis.it

**Keywords:** sarcopenia, diabetes mellitus, obesity, aging, physical exercise, protein supplementation, vitamin D, glucagon-like peptide 1, irisin, myostatin

## Abstract

Sarcopenia is an age-related clinical complaint characterized by the progressive deterioration of skeletal muscle mass and strength over time. Type 2 diabetes (T2D) is associated with faster and more relevant skeletal muscle impairment. Both conditions influence each other, leading to negative consequences on glycemic control, cardiovascular risk, general health status, risk of falls, frailty, overall quality of life, and mortality. PubMed/Medline, Scopus, Web of Science, and Google Scholar were searched for research articles, scientific reports, observational studies, clinical trials, narrative and systematic reviews, and meta-analyses to review the evidence on the pathophysiology of di-abetes-induced sarcopenia, its relevance in terms of glucose control and diabetes-related outcomes, and diagnostic and therapeutic challenges. The review comprehensively addresses key elements for the clinical definition and diagnostic criteria of sarcopenia, the pathophysiological correlation be-tween T2D, sarcopenia, and related outcomes, a critical review of the role of antihyperglycemic treatment on skeletal muscle health, and perspectives on the role of specific treatment targeting myokine signaling pathways involved in glucose control and the regulation of skeletal muscle metabolism and trophism. Prompt diagnosis and adequate management, including lifestyle inter-vention, health diet programs, micronutrient supplementation, physical exercise, and pharmaco-logical treatment, are needed to prevent or delay skeletal muscle deterioration in T2D.

## 1. Introduction

Sarcopenia is defined as an age-related impairment of skeletal muscle performance, resulting in progressive deterioration of mobility, increased risk of falls and fractures, impaired ability to carry out daily activities [1]. According to the European Working Group on Sarcopenia in Older People, sarcopenia may occur with one or more three specific criteria: (a) low muscle strength, (b) low muscle quantity or quality, and (c) low physical performance [2]. Sarcopenia should be suspected if one criterion is satisfied. Two criteria confirm the diagnosis, and three define “severe sarcopenia”. Similarly, according to the Third National Health and Nutrition Examination Survey, muscle mass should be assessed by bioimpedance analysis and be expressed as skeletal muscle index (skeletal muscle mass-to-body mass index × 100). Sarcopenia is defined when individual skeletal muscle index is lower than one standard deviation compared to reference values [3].

Type 2 diabetes mellitus (T2D) is a chronic multifactorial and systemic disease characterized by hyperglycemia and hyperglycemia-induced deterioration of microcirculation and macrovascular complications. The prevalence of T2D increases with age; hence, an overlap between T2D and sarcopenia is anticipated.

Sarcopenia is more prevalent in patients with chronic diseases, such as T2D, indicating that the age-related decline in skeletal muscle performance is faster than in healthy individuals [4,5]. Poor glucose control, longer diabetes evolution, and the presence of chronic diabetes-related complications also increase the risk of sarcopenia in T2D [6,7,8,9].

This review describes the pathophysiological relationship between T2D, sarcopenia, and related outcomes, a critical reexamination of the effect of pharmacological and non-pharmacological interventions in T2D on skeletal muscle health, and a perspective on the myokine signaling pathways involved in glucose control and skeletal muscle metabolism and trophism.

## 2. Methods

PubMed/Medline, Scopus, Web of Science, and Google Scholar were searched for research articles, scientific reports, observational studies, clinical trials, narrative and systematic reviews, and meta-analyses. Appropriate keywords or medical subject headings used in the research strings were as follows: “sarcopenia”; “diabetes mellitus/type 2 diabetes mellitus/T2D”; “aging”; “physical activity/physical exercise”; “protein supplementatio”; “vitamin D”; “antihyperglycemic agent*”; “biguanide”; “dipeptidyl peptidase type IV inhibitor*/DPPIV inhibitor*”; “sodium-glucose transporter type 2 inhibitor*/SGLT2 inhibitor*”; “glucagon-like peptide one receptor agonist*/GLP-1RA”; “thiazolidinedione*”; “insulin analog*”; “acarbose”; “myokine,” “irisin”, “myostatin”.

## 3. Mechanism of Diabetes-Induced Sarcopenia

Evidence suggests that insulin resistance is associated with impaired skeletal muscle glucose uptake and utilization and intracellular accumulation of triglycerides/fatty acids, both associated with sarcopenia [10]. Lipid accumulation in myocytes further reduces skeletal muscle sensitivity to insulin [11]. Insulin resistance, hyperglycemia, and T2D per se induce mitochondrial dysfunction, impaired oxidative metabolism, and energetic utilization, contributing to sarcopenia [12]. In addition, insulin resistance impairs post-prandial myofibrillar protein synthesis due to an imbalance between catabolic and anabolic stimuli at the skeletal muscle site [13].

Proinflammatory cytokines, such as interleukin 1b (IL1b) and tumor necrosis factor α (TNFα), exacerbate protein imbalance by acting as catabolic stimuli [9]. Circulating levels of these cytokines are elevated in T2D and related co-morbidities, thus contributing to background systemic inflammation [14]. Resident macrophages are known to induce and sustain inflammation in the adipose tissue, pancreatic islets, liver, and other peripheral tissues [10], with a significant contribution to the pathophysiology of T2D. As another mechanism, proinflammatory macrophages promote lipolysis, thus exacerbating skeletal muscle steatosis and insulin resistance [12]. Advanced glycation end products (AGEs) also contribute to systemic inflammation and sarcopenia. The effect is attributable to the AGE-mediated activation of scavenger receptors (RAGEs), leading to the activation of proinflammatory pathways associated with systemic inflammation (NF-kB) and oxidative stress (NADPH oxidase) [15]. Hence, background systemic inflammation fosters sarcopenia in patients with T2D and related co-morbidities.

Gut dysbiosis plays a role in chronic intestinal and systemic diseases, including T2D. Notably, the balance between Bifidobacteria and Bacteroides is crucial in maintaining a healthy intestinal barrier. Children develop the best composition of the gut microbiome when vaginally born and breastfed with maternal milk. Once solid foods are consumed daily, Bacteroides and Firmicutes become the prevalent species [16]. Subsequent changes in microbiome composition are related to genetic predisposition and diet. A hypercaloric Western diet predisposes one to T2D and cardiometabolic complications and is characterized by a significant shift in microbiome composition, expressed as a high Bacteroides to Bifidobacterial ratio. The mechanisms explaining the relation between gut dysbiosis and T2D are related to a significant change in intestinal mucosal membrane permeability that facilitates bacterial leakage and translocation from the gut lumen to the subepithelial space, eventually promoting endotoxemia, systemic inflammation, impaired insulin synthesis, and insulin resistance [17]. In addition, some specific species, such as *Akkermansia muciniphila*, are also less expressed in the gut of individuals with T2D, and this phenomenon is associated with impaired intestinal membrane permeability due to reduced synthesis of mucins, exacerbating bacterial leakage as mentioned above [18]. Moreover, gut dysbiosis is associated with defective synthesis of essential micronutrients, such as vitamin B12 and tryptophan, that play a crucial role in skeletal muscle homeostasis, with the latter phenomenon explaining the role of gut dysbiosis in T2D and sarcopenia [19].

Suboptimal chronic protein intake is an age-related nutritional concern. Several factors influence protein intake with advancing age, including physiological changes, such as reduced daily energy requirement, genetic predispositions to low appetite, dental issues, impaired gastric acid secretion and slow gastric emptying, pathological conditions, including physical and mental disabilities, inability to prepare or consume food, dysphagia, and environmental factors such as financial concerns or loneliness [20]. Moreover, these background conditions also affect bromatological diet composition in favor of carbohydrates (and rapidly adsorbed carbohydrates), thus increasing the risk of T2D, obesity, and sarcopenia.

Vitamin D (Vit-D) deficiency and insufficiency are frequently observed in old people. The leading causes of the age-related fall in Vit-D levels are attributable to low intake of naturally Vit-D-rich foods (e.g., meats, fish, eggs, milk, and milk-derived foods) and impaired dermal Vit-D metabolism. Vit-D deficiency is a usual finding in T2D. Vit-D deficiency contributes to impaired insulin synthesis and insulin resistance, increasing the risk of prediabetes and T2D [21]. Vit-D deficiency also contributes to sarcopenia, osteomalacia, osteoporosis, and the risk of falls and fractures [22].

**Figure 1:**
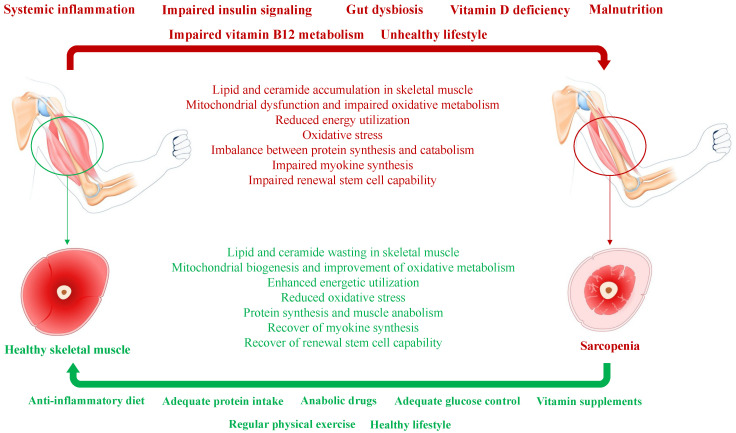
Simplified pathogenesis of diabetes-related sarcopenia and reverting mechanisms involved in the switch from sarcopenia to healthy skeletal muscle.

Hormonal changes occur along with aging. The decline in the frequency and amplitude of growth hormone (GH) peaks and insulin-like growth factor (IGF) 1 is observed in old patients [23]. A similar imbalance is also known for testosterone in men and estrogen, progesterone, and ovarian- and adrenal-derived androgens in women [24,25]. T2D is frequently associated with male hypogonadism, with both conditions fostering sarcopenia in affected men [26]. Figure 1 depicts the pathogenesis of diabetes-related sarcopenia and the potential mechanisms to restore healthy skeletal muscle from sarcopenia.

T2D is associated with insulin resistance, chronic (low-grade) systemic inflammation, unhealthy lifestyle, malnutrition, and microbiome changes that represent concurrent factors of sarcopenia (indicated in bold red above the horizontal red arrow). Concurrent factors induce a significant perturbation in the physiological functions and biochemical activities of skeletal muscle (shown in red below the horizontal red arrow). A healthy lifestyle, including diet, protein and vitamin supplementation, regular physical exercise, and anabolic supplementation when necessary (indicated in bold green below the horizontal green arrow), attenuates skeletal muscle catabolism and may revert sarcopenia to healthy skeletal muscle (shown in green above the horizontal green arrow).

## 4. Sarcopenic Obesity

The term sarcopenic obesity defines a chronic condition in which obesity, T2D, and sarcopenia coexist. Sarcopenic obesity, compared to obesity alone, negatively affects the quality of life and increases the risk of cardiometabolic disorders and overall mortality [27].

It has been estimated that around 30% of older Italian patients diagnosed with sarcopenia had a concomitant condition of sarcopenic obesity, and T2D increased the risk of sarcopenic obesity by 73% [28]. According to the New Mexico Aging Process Study [29], sarcopenic obesity is diagnosed when skeletal muscle mass is at least two standard deviations below the mean reference value for weight-normalized skeletal muscle mass, i.e., <7.26 kg/m^2^ in men and <5.45 kg/m^2^ in women, and body fat mass is greater than 27% in men and 38% in women. According to the Third National Health and Nutrition Examination Survey [30], sarcopenic obesity occurs when skeletal muscle mass is less than 9.12 kg/m^2^ in men and <6.53 kg/m^2^ in women and fat mass >37.16% in men and >40% in women.

The mechanisms involved in sarcopenic obesity are similar to those described for sarcopenia. Apart from insulin resistance, systemic inflammation, physical inactivity, and malnutrition, patients with sarcopenic obesity usually display marked hormonal impairment. Low circulating levels and marked impairment of liver sensitivity to GH and, consequently, low circulating levels of IGF 1 have been reported, as well as functional hypogonadism in men [31].

In addition, dysfunctional skeletal muscle to adipose tissue crosstalk is involved in the pathogenesis of sarcopenic obesity. Interleukin 6 (IL6) is secreted by several types of cells, including striate myocytes. In healthy individuals, skeletal muscle activation leads to an acute increase in circulating levels of IL6 during and hours after the conclusion of a bout of exercise. This sharp rise in IL6 is not detrimental. Instead, it is followed by an improvement in insulin sensitivity, which in turn facilitates glucose uptake and protein synthesis in skeletal muscle [32]. Conversely, chronic overexposure to IL6 due to systemic inflammation fosters T2D and sarcopenia.

## 5. Sarcopenia: A Determinant of Glucose Deterioration and Poor Outcomes

Sarcopenia is associated with low glucose disposal at the skeletal muscle site [33]. Skeletal muscle is responsible for around 80% of glucose uptake during experimental conditions of euglycemic hyperinsulinemic clamp [34]. Skeletal muscle serves as a sort of buffer against hyperglycemia after a glucose load, as observed in the post-prandial phase under physiological conditions [35]. Preserving skeletal muscle mass prevents the onset of prediabetes and progression to T2D [34], as healthy insulin-sensitive skeletal muscle is essential to regulate glucose disposal. First, insulin stimulates the endothelial expression of nitric oxide synthase, nitric oxide production, and peripheral vasodilation. This mechanism ensures adequate blood flow and nutrient supply to skeletal muscle. Second, insulin stimulates the Akt/PKB-mediated translocation of glucose transporters, such as GLUT4, on myocyte membranes. Therefore, insulin is essential in increasing overall glucose uptake in skeletal muscle [36]. The third mechanism is insulin-independent and involves an extracellular matrix interposing the space between microvascular vessels and myocytes. In T2D, myocyte steatosis prompts insulin resistance, oxidative stress, cell injury, necrosis, and apoptosis. All these events stimulate the recruitment and translocation of peripheral monocyte/macrophage-derived proinflammatory cells into skeletal muscles, resulting in local inflammation, accumulation of cellular debris, and fibrillar amorphous matrix. The extracellular matrix becomes a thicker tissue, hindering glucose transport from vessels to myocytes. Inflammation, insulin resistance, and impaired regulation of intramuscular blood flow significantly affect glucose disposal by skeletal muscle [37].

Myokines are a group of proteins with autocrine, paracrine, and endocrine activities, and are produced and released by myocytes, whose expression increases with healthy skeletal muscle [38,39]. These molecules control muscle metabolism and growth, and have immunoregulatory effects [40]. Myokines can be classified as positive and negative regulators of muscle growth, differentiation, and repair. Bone-morphogenic proteins and irisin are the leading positive regulators, along with follistatin, which is secreted at the liver site. Myostatin, transforming growth factor β, activins, and growth differentiation factor are the foremost negative regulators [41,42]. A negative balance between myokines affects the differentiation, proliferation, and repair of myocytes and impairs myofibrillar synthesis, leading to sarcopenia [43]. Myokines, such as IL6, IL10, IL15, irisin, myonectin, osteocorin, and secreted proteins acidic and rich in cysteine (SPARC), are also involved in the crosstalk between skeletal muscle and peripheral tissues, such as pancreatic islets, the liver, adipose tissue, and in the regulation of insulin sensitivity, glucose metabolism, metabolite utilization, and energy expenditure [44,45,46].

Physical inactivity and a sedentary lifestyle are associated with insulin resistance, poor glucose control, and metabolically related consequences, including metabolic syndrome, T2D, obesity, and cardiovascular diseases [47,48]. A sedentary lifestyle is associated with loss of mechanical stimuli, consequent impairment of skeletal muscle trophism [49], skeletal muscle loss [50], and impaired myokine secretion. All these events are involved in impaired glucose metabolism and T2D pathophysiology. Overall, sarcopenia is an independent risk factor of new-onset T2D in normal-weight older people [51], as sarcopenic compared to non-sarcopenic patients require frequently a multipharmacological approach to manage chronic diseases [52] and related outcomes [53].

## 6. Modifiable Risk Factors

Aging is the foremost non-modifiable risk factor for sarcopenia and T2D. However, it is not the only risk factor associated with skeletal muscle health deterioration. Physical exercise, education about a healthy lifestyle, and social status are important and potentially modifiable risk factors of both sarcopenia [54,55,56] and T2D [57]. Adequate management of modifiable risk factors has positive consequences in the prevention and treatment of sarcopenia and T2D in the general population. Nonetheless, managing these non-modifiable risk factors in a community-based manner takes work, especially considering that current healthcare policies usually focus on individually centered management of patients. In addition, healthcare policies do not routinely supply healthcare facilities with sufficient time, space, adequate specialists, and appropriate technological support to improve the quality and efficacy of prescriptions for lifestyle changes, especially exercise programs [58]. Consequently, most patients usually receive less specific advice without supervision on lifestyle adjustments. This approach is characterized by a considerable heterogeneity in the results because of patients’ background differences, such as financial resources and the possibility of adequate access to care. Keeping in mind the current limitations and given the importance of structured and supervised lifestyle change interventions, health policies should endorse more education and specific psychosocial and financial support to facilitate adherence to interventions for most.

## 7. Preventing Sarcopenia: A Therapeutic Target in Primary and Secondary Prevention of T2D

### 7.1. The Physiological Role of Healthy Skeletal Muscle in Preventing Sarcopenia and Glucose Metabolism Deterioration

Preserving myokine syntheses, such as irisin, IL6, myonectin, decorin, fibroblast growth factor (FGF) 19, IL15, SPARC, and brain-derived neurotrophic factor or BDNF, results in a significant improvement in mitochondrial function and skeletal muscle metabolism, myofibrillar synthesis and skeletal muscle growth, insulin secretion, peripheral glucose and lipid utilization, with overall improvement in body composition (including fat mass loss) [59,60,61]. Moreover, suppressing the synthesis of myostatin, a potent skeletal muscle-derived transforming factor that acts as an endogenous inhibitor of myofibrillar synthesis and muscle growth, or increasing the hepatic synthesis of follistatin, a potent endogen myostatin inhibitor, can be additional strategies to improve muscle trophism. High circulating myostatin levels are observed in sarcopenic patients, in whom myostatin concentration is inversely related to follistatin, GH, IFG 1, testosterone, and estradiol [62]. Physical exercise, directly or through indirect metabolic changes, such as low insulin-to-glucagon ratio, GH, and IGF1, enhances the synthesis of myokines and promotes the liver-mediated secretion of follistatin with a net effect on skeletal muscle gain [63,64].

Satellite cells are muscle-derived stem cells that play an essential role in skeletal muscle repair and regeneration [65]. Preserving satellite cells would result in an antiaging effect, an important ally against sarcopenia.

Suppressing the adenosine monophosphate-activate protein kinase (AMPK)–mammalian target of rapamycin (mTOR) pathway by the TGF-β-small mother against decapentaplegic (Smad) signaling results in impaired muscle synthesis and muscle atrophy [66,67]. Therefore, reinforcing the mTOR pathway is beneficial for skeletal muscle health.

Testosterone, estradiol, and GH and IGF1 provide essential anabolic stimuli to increase skeletal muscle mass and reverse skeletal muscle impairment [68]. As the efficiency of the hypothalamus–pituitary–gonadal and GH-IFG-1 axes declines considerably over time, skeletal muscle trophism is significantly impaired by aging. Anabolic hormones are dampened in T2D, especially functional male hypogonadism, with both conditions considered significant contributors to sarcopenia [24].

### 7.2. Non-Pharmacological Intervention: The Role of Lifestyle Changes and Supplements

Evidence suggests that adequate protein intake, monounsaturated acid supplementation, and anti-inflammatory diets have a therapeutical potential to improve muscle health and prevent sarcopenia [69,70,71,72,73] (Table 1). Ramified aminoacidic supplementation attenuates skeletal muscle catabolism and induces skeletal muscle mass gain when combined with regular training, especially resistance training [74,75]. Generally, dietary intervention and physical exercise improve body composition and skeletal muscle strength at all ages [76,77].

Most diet protocols have been demonstrated to affect testosterone synthesis in men. Intermittent fasting protocols are associated with serum total testosterone decline. A reduction in serum testosterone is usually not associated with short-term lean and skeletal muscle mass and loss of strength [78], even though more study is needed to clarify the long-term effects of such protocols. Low-fat diets are associated with significant weight loss and improvement in insulin sensitivity but are also associated with a considerable decline in testosterone concentration with potentially adverse effects on lean mass and body composition [79]. A mild but significative reduction in serum testosterone has also been observed with the Mediterranean diet [80]. Conversely, a low-carb diet with moderate-to-high protein intake not exceeding 3.4 g/kg/day is usually associated with a neutral or even ameliorating effect on serum testosterone [81,82,83]. Very low-carb diets induce a significant increase in serum testosterone, even if the magnitude of this effect is strictly associated with weight loss and the patient’s age [84].

Estradiol is also essential for skeletal muscle health [85]. Phytoestrogens, polyphenols, and hormonal replacement therapy can be considered in post-menopausal women to reinforce muscle health [86,87].

Vit-D is essential for skeletal muscle health [88]. Low circulating levels of 25OH-Vit-D were found in sarcopenic compared to healthy individuals [89]. Vit-D supplementation is associated with gain in muscle strength and, possibly, skeletal muscle mass in healthy and sarcopenic people [90,91,92]. Combining resistance exercise with adequate protein intake and Vit-D supplementation ensures better results on skeletal muscle performance in sarcopenic people [93,94]. Vit-D supplementation is also proven to boost testosterone synthesis in men. Vit-D receptors were found in testicular tissue, especially Leydig cells, where the vitamin is locally activated [95] and stimulates the synthesis of testosterone [96,97]. Men with Vit-D deficiency and insufficiency display reduced levels of serum testosterone and lower testosterone-to-luteinizing hormone ratio, indicating that sufficient exposure to vitamin D is required to sustain the testicular synthesis of testosterone [98]. Vit-D deficiency and male hypogonadism usually coexist and are both independent risk factors for frailty [99]. On the other hand, testosterone affects the peripheral metabolism of Vit-D by enhancing the synthesis of 1,25OH-Vit-D, such as in the kidneys, intestine, and bone tissue [100,101]. This mechanism is probably an additional contributor to the pathogenesis of osteopenia/osteoporosis in hypogonadal men [95]. Compared to standard supplementation of VIT-D (800–1000 IU/day), high-dose Vit-D (>3000 IU/day per 1 year) increases circulating levels of testosterone in healthy individuals [102,103]. Primitive testicular damage is associated with impaired testosterone synthesis and 1,25OH-hydroxylase activity [104]; therefore, sufficient levels of active Vit-D (calcitriol) are required to stimulate testosterone synthesis [105].

### 7.3. Pharmacological Intervention

#### 7.3.1. Biguanides

Metformin, a biguanide approved for T2D, is widely used as a first-line treatment of T2D [106]. Metformin induces controversial results on body composition, skeletal muscle health, and strength. On the one hand, metformin suppresses hepatic glucose output and improves glucose metabolism in skeletal muscle, consequently ameliorating energy utilization and preventing muscle steatosis, two key biochemical elements to prevent or treat sarcopenia [107]. Metformin also exhibits anti-inflammatory and antioxidative properties, improves satellite cell viability and regenerative effects, and promotes myofibrillar repair [108,109,110]. The mechanism by which metformin improves muscle repair could be attributable to the drug-induced attenuation of Smad 2 and 3 activities in the context of the TGF-β signaling pathway [111], as the attenuation of this pathway stimulates insulin secretion and myofibrillar synthesis [112,113]. On the other hand, metformin affects the AMPK/mTORc1 pathway, thus reducing glucose output from the liver and fasting glucose levels. The inhibition of mTORc1 is also associated with impaired protein synthesis and autophagy, which results in defective myofibrillar synthesis and skeletal muscle hypotrophy [114]. Moreover, metformin activates the forkhead box O3a or FoxO3a, via AMPK, a key transcription factor of myostatin, a potent inhibitor of myofibrillar synthesis and skeletal muscle growth [115]. Observational data suggest that metformin may have a protective effect against sarcopenia [116,117,118]. However, the level of evidence is slight and possibly affected by confounding factors, such as exercise, diets, or nutraceuticals (Table 2).

#### 7.3.2. Secretagogues

Sulfonylureas have been widely used for treating hyperglycemia in T2D. The drugs bind to a specific site of the ATP-sensitive K-channel in the β-cell plasma membrane and close it, consequently blocking the potassium outflow, depolarizing the cell membrane, and initiating the signal cascade, eventually resulting in insulin release [119]. Sulfonylureas potentiate glucose disposal in skeletal muscles [120]. Nevertheless, preclinical studies have found that inhibiting ATP-sensitive potassium channels is associated with muscle atrophy. In addition, sulfonylureas may enhance caspase-3 activity and reduce the protein content in skeletal muscle [121]. Overall, these data indicate that sulfonylureas have detrimental effect on skeletal muscle health.

#### 7.3.3. Intestinal Glucosidase Inhibitor

Acarbose, an α-glucosidase inhibitor, is an antihyperglycemic agent able to attenuate post-prandial glycemic excursion after food intake [122]. Data suggest that acarbose could be associated with impaired muscle trophism and strength, with unclear mechanisms [123]. Caution should be taken while prescribing α-glucosidase inhibitors in patients at risk for or diagnosed with sarcopenia [124].

#### 7.3.4. Dipeptidyl Peptidase Type IV Inhibitors

Dipeptidyl peptidase type IV inhibitors (DPPIVis) belong to the incretin family, a class of drugs affecting the incretin system. DPPIVis compete with endogen incretins, such as the glucose-dependent insulinotropic polypeptide (GIP) and glucagon-like peptide 1 (GLP-1), to the catalytic site of the enzyme and inhibit their degradation [125]. Therefore, DPPIVis improve glucose control by extending the circulating half-life of endogenous incretins, especially in the post-prandial phase [126]. Besides their well-established effect on glucose control in terms of absolute efficacy and durability [127], DPPIVis are associated with a neutral effect on body weight and composition [128]. However, mechanistic data suggest that DPPIVis may sustain skeletal muscle trophism. First, they have the potential to regulate the arteriolar diameter and, consequently, increase blood flow in skeletal muscle [129]. Second, they enhance the insulin-mediated translocation of glucose transporters on the myocyte surface [130], have antioxidative and anti-inflammatory effects, and improve mitochondrial function and oxidative phosphorylation [131]. The latter effect could be mediated by DPPIV-induced sympathetic activation rather than a direct effect, as observed in the post-prandial phase in T2D individuals [132]. DPPIVis improve exercise tolerance in patients with heart failure by stimulating mitochondrial biogenesis in skeletal muscles [133,134]. The peroxisome proliferator co-activator 1 alpha (PGC-1α) plays a direct role in mitochondrial biogenesis and mitophagy, two fundamental biological events related to mitochondrial viability and function [135]. Notably, PGC-1α is downregulated in the skeletal muscle of patients with T2D and DPPIVis, such as sitagliptin, stimulate the PGC-1α synthesis [136] with protective effects against insulin resistance, skeletal muscle hypotrophy, and impaired glucose and lipid metabolism [137]. Overall, clinical data indicate that DPPIVis do not improve skeletal muscle mass or cardiometabolic fitness, with an uncertain effect on sarcopenia [138].

#### 7.3.5. Thiazolidinediones

Thiazolidinediones are insulin-sensitizer agents as they increase significantly glucose uptake in skeletal muscle at rest and after exercise [139]. Specifically, thiazolidinediones increase the phosphorylation of protein kinase B and insulin-stimulated phosphoinositide 3-kinase activity in skeletal muscle [140,141]. This insulin-sensitizing effect is also mediated by an increase in serum adiponectin concentration and adiponectin receptor 1 in skeletal muscle and adipose tissue, which is associated with enhanced glucose uptake [142,143]. Moreover, thiazolidinediones are well-known peroxisome proliferator-activated receptors γ which regulate adipocyte differentiation, fatty acid storage, and glucose metabolism [144]. Thiazolidinediones affect lipid metabolism by increasing HDL, decreasing LDL and triglycerides, and attenuating liver steatosis, which is associated with sarcopenia [145,146]. As demonstrated in mouse models, the fructose transporter GLUT5 is markedly enhanced in skeletal muscle. It is a possible adaptive mechanism to overcome an impaired glucose metabolism, but it predisposes to steatosis [147]. Rosiglitazone reduces the expression of GLUT5, hence playing a protective role in both the liver and skeletal muscle. Pioglitazone has anti-inflammatory properties in skeletal muscle [148] and improves mitochondrial function in T2D [149]. Moreover, thiazolidinediones reduce circulating and intramuscular levels of ceramides [150], which play a pathophysiological role in insulin resistance in skeletal muscle and accentuate the risk of age-related sarcopenia in T2D [151]. Clinical data indicate that pioglitazone increases whole-body aerobic capacity and skeletal muscle energy metabolism, thus providing beneficial effects on muscle trophism and physical performance [152]. Because of the adipogenic potential of thiazolidinediones, fat mass gain at the subcutaneous but not visceral adipose tissue site [153,154] is a common finding in T2D. Moreover, pioglitazone stimulates the commitment of skeletal muscle satellite cells to adipocytes [155]. Nevertheless, these phenomena are not associated with skeletal muscle impairment [156]. Thiazolidinediones significantly increase total body water content, as they stimulate sodium retention. However, they did not affect the level of skeletal muscle hydration [157]. A decline in bone mineral density has also been reported in patients on thiazolidinediones [158]. A few cases in the literature of thiazolidinedione-induced rhabdomyolysis have been described. The mechanisms are unclear, also considering that neither statins nor physical exercise were ascertained as promoting factors [159]. Besides sporadic cases, thiazolidinediones should be considered safe and possibly effective medications for preserving muscle health in T2D.

#### 7.3.6. Gliflozins

Gliflozins or sodium-glucose (co)transporter type 2 inhibitors (SGLT2is) act as antihyperglycemic agents by blunting glucose resorption at the proximal renal tubule site through an insulin-independent mechanism [160]. SGLT2is reduce glucose resorption by 30 to 50% [161] and are thus responsible for a moderate but significant caloric dissipation of approximately 200 Kcal/day [162]. Glycosuria is also responsible for osmotic diuresis, which leads to transient extracellular water and sodium depletion [163]. SGLT2is are associated with broad positive effects beyond glucose control, including a mild reduction in blood pressure and weight loss and cardiovascular and renal benefits. Moreover, SGLT2is have antioxidative effects, improve mitochondrial function, provide a favorable metabolic shift towards fatty acids and ketone bodies rather than glucose in myocytes, stimulate erythropoiesis, attenuate the sympathetic tone [164], insulin resistance, and systemic inflammation [165]. These effects ameliorate cardiac pump efficiency and improve long-term outcomes related to chronic heart failure, regardless of hyperglycemia and background cardiac pump efficiency [166]. SGLT2is affect body composition by specifically reducing both fat and fat-free mass [167]. Despite causing a significant reduction in fat mass in both subcutaneous and visceral adipose areas, these medications have had some controversial results [168], including increasing the long-term loss of lean mass and skeletal muscle mass [169]. Because of this phenomenon, physical exercise, diet, and an adequate protein intake are needed to minimize muscle mass impairment in T2D patients on SGLT2is [170]. On the other hand, SGLT2is have been demonstrated to improve skeletal muscle performance in patients with T2D and heart failure. Low cardiac output, tissue hypoxia, hormonal and metabolic imbalance, and forced inactivity or immobilization are the leading mechanisms explaining skeletal muscle deterioration in T2D with heart failure [171]. As SGLT2is improve cardiac function, tissue perfusion, and oxygenation and provide a metabolic boost to skeletal muscle mass metabolism [172], this class of medications may be helpful in preserving skeletal muscle mass and performance in T2D with chronic heart failure [173].

#### 7.3.7. Glucagon-like Peptide 1 Receptor Agonists

Glucagon-like peptide 1 receptor agonists (GLP-1RAs) belong to the incretin class approved for T2D. Apart from semaglutide, which includes injectable and oral formulations, GLP-1RAs are administered subcutaneously with simple-to-use devices. Clinical trials showed that GLP-1RAs, especially long-acting analogs, are potent antihyperglycemic agents and induce significant weight loss without affecting the risk of hypoglycemia [174]. GLP-1RAs work in several domains. The leading therapeutic effects of GLP-1RAs include the enhancement of glucose-dependent insulin release and suppression of glucagon secretion during hyperglycemia, but not hypoglycemia, delay of gastric emptying, and suppression of appetite [175,176]. GLP-1RAs also provide cardiovascular and renal protection. As GLP-1RAs work efficaciously and safely, the prescription of this class of medications has gained success in T2D, even compared to other equally potent but more expensive and composite regimens, such as insulin therapy [177,178,179]. GLP-1RAs are expected to affect body composition significantly. Patients on GLP-1RAs lose weight, fat mass, and visceral adipose tissue instead of fat-free and skeletal muscle masses [180,181,182]. Real-life studies have confirmed previous findings in T2D patients with and without weight excess up to one year of treatment [183,184,185]. Interestingly, GLP-1RAs improve endothelial function in T2D with indirect (improving glucose control and insulin signaling) and direct (receptor agonism) mechanisms, thus improving muscle perfusion and angiogenesis [186]. Some uncertainty remains on the role of GLP-1RAs in modulating skeletal muscle viability and metabolism, since previous evidence did not confirm the presence of GLP-1 receptors on skeletal muscle myocytes. One study found that semaglutide inhibited ubiquitin-proteosome-mediated skeletal muscle proteolysis, thus promoting myogenesis in murine myocytes. These effects were attributable to a GLP-1RA-induced decrease in proinflammatory cytokines and oxidative stress, which in turn was associated with the attenuation of ubiquitin-proteasome muscle wasting and increase in the hepatic synthesis of IGF 1 (myogenesis). Nevertheless, semaglutide was found to improve skeletal muscle atrophy by directly stimulating GLP-1 receptors in myocytes by the cAMP-mediated activation of PKA and AKT [187]. Another study found that liraglutide and semaglutide improved glucose tolerance and insulin sensitivity, reduced body weight gain and excessive lipid accumulation, and enhanced muscle atrophy in a high-fat diet model of obesity by activating the SIRT1 pathway [188]. Evidence suggests that GLP-1RAs boost irisin release and reduce IL6 secretion after 6 months of treatment, indicating favorable effects on skeletal muscle and adipose tissue, as both irisin deficiency and chronic exposure to moderate-high levels of IL6 are associated with insulin resistance, impaired insulin secretion, poor glucose control, weight gain, expansion of visceral adipose tissue, and muscle hypotrophy [189,190]. GLP-1RAs also attenuate the expression of atrophic factors in mice, thus decreasing the skeletal muscle catabolism associated with advancing age and T2D [191] and stimulating the expression of antiatrophic factors and differentiation of satellite stem cells to improve skeletal muscle regenerative potential [192]. In addition, GLP-1RAs boost the hypothalamus–pituitary–testicle axis and increase serum testosterone concentration in T2D and functional hypogonadism, positively affecting weight loss and body composition [193,194].

#### 7.3.8. Dual GLP-1/GIP Co-Agonists

Dual GLP-1/GIP co-agonists have recently been approved for T2D [195], and GLP-1/glucagon co-agonists and triple (glucagon, GLP-1, and GIP) agonists are under investigation [196,197]. It has been demonstrated that co-agonists provide a synergic effect on appetite and energy intake compared to GLP-1RAs alone [198]. Clinical trials indicate that dual GLP-1/GIP co-agonists, such as tirzepatide, should be considered the most effective agents compared to other antihyperglycemic drugs in the early stage as well as long-lasting T2D [199,200,201,202,203,204]. Clinical trials and meta-analyses also indicated that tirzepatide, compared to placebo, reduces body weight by 7.5 to 12 kg (5 to 15 mg/weekly) and 1.7 to 7.2 kg compared to GLP-1RAs [205,206]. Ongoing investigations confirm these impressive results on weight loss also in obese individuals. In the SURMOUNT-1 trial, the mean percentage change in weight after 72 weeks of treatment was −15% with 5 mg weekly doses of tirzepatide, −19.5% with 10 mg doses, and −20.9% with 15 mg doses compared to −3.1% with placebo. The number of patients who lost more than 20% of baseline body weight while on 10 mg (50%) and 15 mg doses (57%) was significantly higher compared to placebo (3%) [207]. Slightly lower but significant results were also found in obese individuals with T2D, as demonstrated by the SURMOUNT-2 trial [208]. Tirzepatide in addition to a lifestyle intervention also boosted weight loss in patients who had achieved a satisfactory weight reduction (i.e., ≥5.0%) after a 12-week intensive lifestyle intervention [209]. Overall, the promising results of dual agonists are approaching those obtained with bariatric surgery [210,211], in a magnitude never seen until today [212]. Despite remarkable weight loss, tirzepatide was found to selectively reduce fat, but not free-fat mass, as recently demonstrated [213,214]. Thanks to these results, tirzepatide is expected to prevent and dramatically change the clinical course of cardio-nephron-metabolic diseases [215], but specific trials are needed to ascertain the magnitude of positive effects on skeletal muscle health and strength.

#### 7.3.9. Insulin Analogues

Insulin promotes muscle growth by stimulating myofibrillar synthesis and increasing skeletal muscle blood flow, amino acid delivery, and availability [216]. The leading mechanism by which insulin promotes skeletal muscle protein synthesis and muscle growth is attributable to the activation of the phosphatidylinositol 3-kinase–mTOR pathway, in an opposite way compared to how metformin works [217]. Insulin analogs act exactly like human insulin to stimulate glucose and amino acid uptake in skeletal muscle myocytes [218]. Also, insulin is essential to the promotion of glucose utilization, oxidative mitochondrial respiration, and substrate accumulation after energy replacement in skeletal muscles in a dose-dependent manner [219]. Experimental models suggest that insulin therapy reduces myocyte apoptosis and attenuates skeletal muscle wasting in rats by alleviating reticulum endoplasmic stress [220]. However, long-term insulin treatment was found to produce significant histological changes in skeletal muscle myocytes, including the level of expression of myosin heavy chains, shift toward type II fibers, and reduced expression of several myokines, such as IL6, myostatin, and irisin [221]. Despite the potential for improving skeletal muscle health, these changes are involved in insulin resistance and the deterioration of skeletal muscle performance, which depends on chronic exposure to endogen insulin or exogen analogs and occurs in a dose-dependent manner. Insulin treatment must be adjusted over time, as most of its hypoglycemic potential is obtained when a treat-to-target approach is carried out. The need for intensifying insulin regimens depends, at least in part, on a progressive decline in insulin sensitivity, which, in turn, is related to several factors, including obesity and chronic insulin exposure per se. Progressive titration of insulin analogs leads to a considerable increase in the total daily dose of insulin, which results in weight gain, risk of hypoglycemic events, and further deterioration of skeletal muscle mass and insulin sensitivity. Strategies to improve insulin sensitivity are therefore necessary to attenuate this vicious circle [222] and preserve skeletal muscle health.

Given the pathophysiology of T2D, most patients receive combined treatment in their lifetime to achieve tailored control of glycemic and extra-glycemic targets. Metformin is the background treatment of T2D; adding DPP-IVis, thiazolidinediones, and SGLT2is is expected to improve skeletal muscle health by balancing the positive and potentially detrimental effects of each class of drugs individually. Adding GLP-1RAs or more composite regimens, such as basal insulin or basal-GLP-1RAs, may induce interesting results as well [223].

## 8. Future Directions

Tailored interventions are the new frontiers of precision- and evidence-based medicine. Targeting energy expenditure, fat oxidation, appetite regulation, and lean mass preservation are key elements for sustainable weight loss in metabolic disorders, including T2D [224]. The pharmacological treatment of T2D induces different effects on skeletal muscle health. Metformin, acarbose, and secretagogues do not improve body composition and skeletal muscle mass, as they have neutral or even detrimental effects on muscle health. Thanks to a wide range of metabolic and non-metabolic effects, thiazolidinediones and DPP-IVis have the potential for attenuating age-related skeletal muscle mass decline in T2D and, possibly, in earlier stages of impaired glucose metabolism. However, they have not been demonstrated to increase skeletal muscle mass or strength. Moreover, DPP-IVis do not affect body weight and composition, while thiazolidinediones induce weight and fat mass gain over time. SGLT2is have been demonstrated to improve body composition, especially by providing mild-to-moderate weight loss more than other oral antihyperglycemic agents. Nevertheless, some data suggest that gliflozins might impair muscle mass and strength, leading clinicians to consider appropriate lifestyle adjustments or avoid SGLT2is prescription in patients at risk of or with sarcopenia [225]. However, these agents improve skeletal muscle health, exercise tolerance, and overall physical performance in patients T2D with heart failure. GLP-1RAs have the potential to affect body composition healthier. They reduce body weight, fat mass, and visceral adipose tissue while preserving or even improving skeletal muscle mass and strength regardless of baseline body composition and BMI. Nevertheless, GLP-1RAs significantly reduce food intake and appetite, and their use in sarcopenic patients may be complicated by further weight loss and the inability to consume hypercaloric diets and protein supplementations. Prescribing GLP-1RAs may be, therefore, intricate in sarcopenic patients or those at high risk of sarcopenia. Insulin therapy can potentially improve skeletal muscle mass and induce weight gain. However, it is less likely to be handled easily compared to non-insulin regimens, raises hypoglycemic risk, and requires adequate daily glucose monitoring and proper adherence by patients or caregivers.

Future research is needed to address more evidence on the management of sarcopenia in T2D. Aside from biomechanical function, skeletal muscle has significant endocrine activities. Preserving skeletal muscle endocrine functions means maintaining essential crosstalk between skeletal muscle and several tissues, such as the brain, adipose tissue, bones, the liver, gut microbiome, pancreatic islets, microvasculature, skin, and muscle itself [226]. A few studies have been conducted in the long term, highlighting non-significant or controversial results on skeletal muscle mass and strength [227]. Among antihyperglycemic agents, SGLT2is were found to preserve healthy myokine secretion, which is essential to the maintenance of both metabolic and functional activity of skeletal muscles. Nevertheless, they probably have neutral or detrimental effects on atrophic factors, such as myostatin. As indicated in preclinical studies, GLP-1RAs may induce anabolic stimuli in skeletal muscles and potentiate muscle-regenerative properties. Evidence suggests that combining physical exercise with diet ensures more significant effects on skeletal muscle health. Although similar positive results can be anticipated while considering the combination of physical activity and antihyperglycemic treatments, no specific studies have been carried out to confirm or deny this hypothesis.

Myokine-based therapy has the potential to be a new therapeutic frontier. It is widely accepted that physical exercise has antidegenerative and renewal properties, and myokines are the leading mediators of these beneficial effects. Irisin is primarily involved in maintaining muscle cell and bone health thanks to its anti-inflammatory [228] and regenerative properties [229]. Mechanistic studies have shown a close association between irisin deficiency and the development of insulin resistance and cardiometabolic complications, such as pathological myocardial remodeling [230]. Aerobic, high-intensity interval training and combined aerobic–resistance workouts increase irisin levels considerably in different settings and regardless of background characteristics in terms of cardiorespiratory fitness and weight status [231,232]. Caloric restriction alone seems not to preserve lean mass and slightly reduces irisin concentration [233]. Interestingly, Vit-D supplementation increases the level of circulating irisin by directly stimulating the intramuscular synthesis of its precursor (fibronectin type III domain-containing protein 5 or FNDC5) [234], which probably mediates both metabolic and functional parameters [235,236]. There is a direct relationship between serum testosterone concentration and irisin levels in men with metabolic syndrome [237,238]. Other data show that insulin resistance is associated with increased basal but not post-exercise levels of irisin [239,240], indicating that the condition of irisin resistance may play a role in the early stages of T2D. Testosterone replacement treatment in male functional hypogonadism, including patients with T2D, is associated with a significant increase in irisin concentration [241]. Conversely, peripheral but not intracerebral irisin administration improve testosterone levels and biologically related consequences such as sexual function and spermatogenesis [242,243,244].

Fibroblast growth factor 19 (FGF19) has significant regenerative, metabolic, and anti-inflammatory properties associated with skeletal muscle growth and hypertrophy [245,246,247], while low levels of FGF19 are associated with sarcopenia [248]. FGF19 reverts obesity-induced muscle atrophy and restores irisin levels [249], thus playing a role in improving skeletal muscle health. One study found that short-term administration of FGF19 improved skeletal muscle growth regardless of food intake in mice [250].

Last, myostatin inhibitors and follistatin analogs can improve skeletal muscle mass and strength. Interesting results can be obtained by monoclonal antibodies targeting the myostatin/activin signaling pathway by antagonizing activin type II receptors, which mediate muscle breakdown [251]. Activin type II receptor antagonism is expected to maximize muscle hypertrophy in the presence of chronic muscle training [252]. Moreover, testosterone, estradiol, and GH suppress myostatin synthesis by downregulating gene expression and stimulate the synthesis of follistatin [253,254]. This mechanism is thought to explain, at least in part, the anabolic effect of sexual steroids and GH on skeletal muscle trophism. Although aging is associated with a progressive decline in gonadal and hypophyseal function, no evidence indicates that replacing dysfunctional axes may result in a long-term and safe amelioration of skeletal and muscle endpoints, including muscle strength, prevention of falls, and frailty [24]. Therefore, targeting myostatin with specific monoclonal antibodies may have a therapeutic rationale, as demonstrated by several trials in patients with primitive or secondary myopathies [255,256] and age-related sarcopenia [257].

## 9. Conclusions

The close interconnection between sarcopenia and T2D is well known. Both conditions are expected to increase in prevalence due to the elongation of life expectancy, as aging is one of the leading contributing factors of T2D and sarcopenia.

Identifying patients at risk of or with sarcopenia is essential for individualizing comprehensive therapeutic programs in T2D, including education, lifestyle adjustments, healthy diet, micronutrients, and protein supplements, regular physical exercise, and appropriate pharmacological treatment to remove risk factors, revert skeletal muscle depletion and, possibly, improve skeletal muscle mass and strength.

Preserving skeletal muscle mass and strength positively affects overall physical performance and independence during daily activities. It also holds important endocrine and metabolic mechanisms underlying significant improvements in glucose control, durability of pharmacological effectiveness, prevention of complications, and amelioration of the quality of life in T2D.

From a research viewpoint and in terms of future directions, more evidence is needed to address the role of pharmacological management of T2D on long-term skeletal muscle health. Myokine-based treatment has the potential to improve skeletal muscle health and provide reliable therapeutic strategies to ameliorate glucose control, positively affect body composition, and prevent and treat sarcopenia in T2D.

## Figures and Tables

**Table 1:** Summary of the leading mechanisms by which non-pharmacologic intervention may affect skeletal muscle health in T2D.

Type of Intervention	Possible Positive Effects on Skeletal Muscle	Possible Detrimental Effects on Skeletal Muscle	Overall Effect
Protein supplementation	Attenuates myofibrillar catabolism	-	Prevent sarcopenia
Vitamin D supplementation	Improve insulin sensitivity	-	Prevent sarcopenia
	Boost testosterone synthesis		
	Boost myokine synthesis (e.g., irisin)		
Diets	Insulin-sensitizing effect	Impairment of testosterone synthesis (low-fat diets, intermittent fasting protocols, vegetable-based diets)	Prevent sarcopenia
	Improve glucose utilization		
	Reduce systemic inflammation		
	Prevent muscle steatosis		
	Induce weight loss		
	(Facilitate adherence and resistance to physical exercise)		
Physical exercise (high-intensity more than low-to-moderate intensity)	Insulin-sensitizing effect	-	Improve muscle mass and strength
	Improve glucose utilization		
	Prevent muscle steatosis		
	Induce weight loss		
	Boost testosterone synthesis		
	Boost myokine synthesis		
	Increase myofibrillar synthesis		
	Reduce myofibrillar catabolism		

**Table 2:** Summary of the leading mechanisms by which pharmacologic intervention affects skeletal muscle health in T2D.

Classes of Antihyperglycemic Agents	Possible Positive Effects on Skeletal Muscle	Possible Detrimental Effects on Skeletal Muscle	Overall Effect
Biguanides (e.g., metformin)	Insulin-like sensitizing effect	Proteolytic effect (Inhibition of AMPK/mTORc1 pathway) Stimulate myostatin synthesis (AMPK/FoxO3a transcription factor)	Neutral or favors sarcopenia
	Improve glucose metabolism		
	Ameliorate energy utilization		
	Anti-inflammatory/antioxidative properties		
	Improve satellite cell viability/regenerative effects		
	Antiproteolytic effect		
	(Inhibition of TGF-β/Smad signaling)		
Secretagogues (e.g., sulfonylureas, glinides)	Unclear	Inhibit ATP-sensitive potassium channels	Favors sarcopenia
		(Muscle atrophy)	
		Enhance caspase-3 activity	
		(Apoptosis)	
Thiazolidinediones (e.g., pioglitazone)	Insulin-sensitizing effect	Direct muscle toxicity? (Rhabdomyolysis, rare adverse event)	Neutral
	Improve glucose utilization		
	Prevent muscle steatosis		
Intestinal glucosidase inhibitors (e.g., acarbose)	Unclear	Unclear	Unclear
DPPIVis	Potentiate microvascular supply	Unclear	Neutral
	Insulin-sensitizing effect		
	Improve glucose utilization		
	Antioxidative/anti-inflammatory effects		
	Enhance the synthesis of PGC-1α		
	(Mitochondrial biogenesis)		
SGLT2is	Metabolic shift toward fatty acids and ketones	Clinical evidence of fat-free mass loss	Favors sarcopenia. Prevent sarcopenia in heart failure
	Improve tissue oxygenation		
	Antioxidative/anti-inflammatory effects		
	Improve cardiac pump efficiency		
	Improve exercise tolerance		
	Boost myokine secretion		
GLP-1RAs	Improve glucose utilization	Excessive weight loss Reduce appetite (might hamper sufficient caloric and protein intake)	Prevent sarcopenia or improve skeletal muscle
	Antioxidative/anti-inflammatory effects		
	Stimulate hepatic synthesis of IGF1 (myogenesis)		
	Boost myokine secretion		
	Improve satellite cell viability		
	Improve satellite cell viability/regenerative effects		
	Promote myofiber repair		
	Boost testosterone synthesis		
Insulin analogues	Improve glucose utilization	Long-term, dose-dependent impairment of insulin sensitivity Muscle steatosis Weight gain and hypoglycemia (Facilitate discontinuation of physical exercise and sedentarism)	Unclear
	Antioxidative/anti-inflammatory effects		
	Potentiate microvascular supply		
	Direct stimulation of myofibrillar synthesis		
	Direct inhibition of myofiber proteolysis		

Abbreviations: transforming growth factor β = TGF-β; small mother against decapentaplegic = Smad; adenosine monophosphate-activate protein kinase = AMPK; mammalian target of rapamycin = mTOR; forkhead box O3 = FoxO3; adenosine triphosphate = ATP; peroxisome proliferator co-activator 1 alpha = PGC-1α; insulin-like growth factor 1 = IGF1; dipeptidyl peptidase type IV inhibitors = DPPIVis; glucagon-like peptide 1 receptor agonists = GLP-1RAs; sodium-glucose (co)transporter type 2 inhibitors = SGLT2is.

## Data Availability

Not applicable.

## Abbreviations

AMPK	Adenosine monophosphate-activate protein kinase
DPPIVis	Dipeptidyl peptidase type IV inhibitors
FGF	Fibroblast growth factor
FGF19	Fibroblast growth factor 19
GLP-1	Glucagon-like peptide 1
GLP-1RAs	GLP-1 receptor agonists
GIP	Glucose-dependent insulinotropic polypeptide
GH	Growth hormone
IGF	Insulin-like growth factor
IL	Interleukin
mTOR	Mammalian target of rapamycin
PGC-1α	Peroxisome proliferator co-activator 1 alpha
SPARC	Secreted proteins acidic and rich in cysteine
Smad	Small mother against decapentaplegic
SGLT2is	Sodium-glucose (co) transporter type 2 inhibitors
TNFα	Tumor necrosis factor α
T2D	Type 2 diabetes
Vit-D	Vitamin D
